# Blood phosphorylated tau elevation as a biomarker in immunoglobulin light chain and transthyretin amyloidosis

**DOI:** 10.1038/s41591-026-04272-2

**Published:** 2026-03-11

**Authors:** Stephan A. Kaeser, Stephanie A. Schultz, Anna Hofmann, Lisa M. Häsler, Ying Xu, Marius Lambert, Ulrike Obermüller, Kathrin Brockmann, Johan Bijzet, Hans Nienhuis, Mario Nuvolone, Laura Obici, Giovanni Palladini, Ute Hegenbart, Stefan O. Schönland, Mathias Jucker

**Affiliations:** 1 German Center for Neurodegenerative Diseases (DZNE), Tübingen, Germany; 2 Department of Cellular Neurology, Hertie Institute for Clinical Brain Research, University of Tübingen, Tübingen, Germany; 3 Department of Neurology, Harvard Medical School, Boston, MA USA; 4 Massachusetts General Hospital, Boston, MA USA; 5 Department of Neurodegenerative Diseases, Hertie Institute for Clinical Brain Research, University of Tübingen, Tübingen, Germany; 6 Department of Laboratory Medicine, Groningen Amyloidosis Center of Expertise Groningen, University Medical Center Groningen, Groningen, the Netherlands; 7 Department of Internal Medicine, Groningen Amyloidosis Center of Expertise, University Medical Centre Groningen, Groningen, the Netherlands; 8 Department of Molecular Medicine, University of Pavia, Pavia, Italy; 9 Amyloidosis Research and Treatment Center, IRCCS Fondazione Policlinico San Matteo, Pavia, Italy; 10 Amyloidosis Center, Medical Department V, University Hospital Heidelberg, Heidelberg, Germany

**Keywords:** Neuroscience, Biomarkers

## Abstract

Elevated blood levels of phosphorylated tau (p-tau) are diagnostic of Alzheimer disease and are associated with the deposition of amyloid-β in the cerebral neuropil. Elevated p-tau levels have also been associated with cerebral deposition of Danish amyloid and prion protein amyloid. Here we analyzed p-tau in serum from four different cohorts of people with the most common types of systemic amyloidosis, transthyretin (ATTR) amyloidosis and immunoglobulin light chain (AL) amyloidosis. We found higher levels of serum p-tau181 in the AL and ATTR groups than in controls. Subsequent analyses revealed that these effects were more pronounced in the presence of polyneuropathy (PNP) and in AL compared to ATTR amyloidosis. Individuals with different forms of PNP that were not due to amyloidosis did not exhibit elevated p-tau181 levels. In cases of presymptomatic (genetic) ATTR, p-tau181 levels increased as a function of predicted years from symptom onset. Additional measurement of p-tau217 in one cohort revealed similar increases, and discriminated people with AL and those with ATTR from controls equally as well as p-tau181. These findings suggest that elevated serum p-tau levels are not specific to Alzheimer disease and may also serve as a diagnostic tool of ATTR and AL amyloidosis, with potential utility in distinguishing amyloidosis-related PNP from PNP of other etiologies.

## Abstract (web summary)

Elevated serum levels of phosphorylated tau are not specific to Alzheimer’s disease and may also serve as a diagnostic tool for the most common types of systemic amyloidosis, with potential utility in distinguishing amyloidosis-related polyneuropathy from polyneuropathy of other etiologies.

## Main

Amyloidoses are protein misfolding diseases in which the misfolded proteins acquire characteristic amyloid fibrils at the ultrastructural level^1^. The most prominent cerebral amyloidosis is Alzheimer disease (AD) with deposition of amyloid-β (Aβ) in the cerebral neuropil. The most common systemic amyloidoses, AL amyloidosis and ATTR amyloidosis, affect a variety of peripheral organs, often concomitant with a progressive PNP^2,3^.

In ATTR amyloidosis, fibril accumulation results from mutations in the transthyretin (*TTR*) gene or accumulation of misfolded wild-type TTR, causing fibril formation and toxic effects on tissues^3,4^. AL amyloidosis is driven by clonal proliferation of plasma cells producing abnormal amounts of immunoglobulin light chains, which misfold and aggregate into amyloid fibrils^5^. Although AL amyloidosis is relatively rare, ATTR, especially the wild-type form, is common in older adults (10–25% in those aged >80 years)^2,3^. The diagnosis of AL and ATTR is complex and stepwise, depending on the affected organs. Renal (serum creatinine, proteinuria) and cardiac (NT-proBNP and troponin) markers are used in disease staging and patient stratification. However, a tissue biopsy or at least a bone scintigraphy (for ATTR) is necessary for definite diagnosis. Thus, there is an urgent need for blood-based AL and ATTR diagnostic biomarkers.

Phosphorylated tau species in cerebrospinal fluid (CSF) and blood are linked to the deposition of Aβ, prion protein amyloid (APrP) and Danish amyloid (ADan) in the brain. Specifically, levels of tau phosphorylated at position 181 (p-tau181) and position 217 (p-tau217) increase in CSF and blood one to two decades before the onset of AD clinical symptoms^6,7^. Both p-tau181 and p-tau217 also increase in the CSF and blood of Creutzfeldt–Jakob disease^8,9^ and are increased in mouse models with brain Aβ or ADan deposition in the absence of tau pathology^10^. These observations suggest that elevated soluble p-tau may result from neuropil changes to any brain amyloid deposition.

To test the hypothesis that the increase in soluble p-tau is an even more universal response of (nerve) cells to amyloid deposition and to search for blood-based biomarkers for AL and ATTR, we studied p-tau levels in the blood of patients with systemic AL and both wild-type and variant ATTR amyloidosis, with or without PNP, and controls.

## Results

**Fig. 1:**
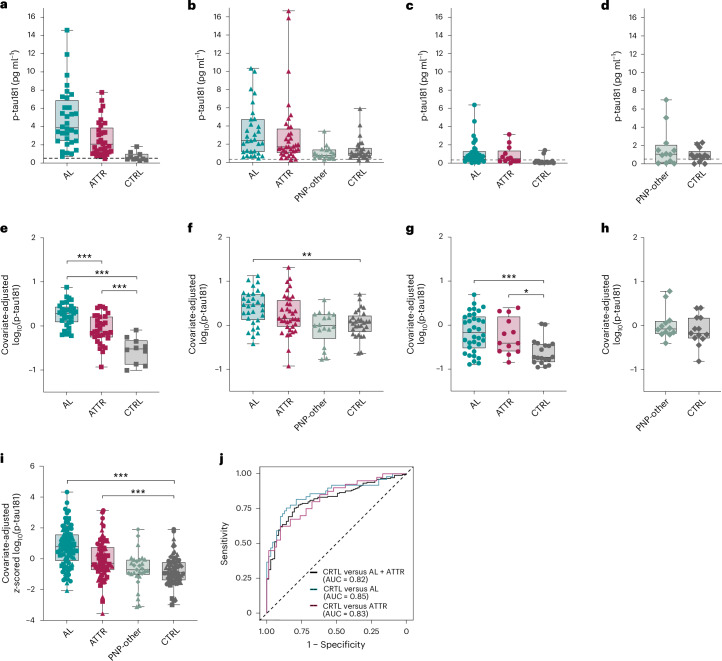
Serum p-tau181 levels in AL and ATTR groups from four different cohorts. **a**–**d**, Absolute (nontransformed) serum p-tau181 levels in patients with AL and those with ATTR from the Pavia (**a**), Heidelberg (**b**), Groningen (**c**) and Tübingen (**d**) cohorts. Patient characteristics and the numbers of patients are given in Extended Data Table 1. Patients were grouped based on disease status. For each cohort, the lower functional limit of quantification (LLoQ) is indicated by a gray dashed line. **e**–**h**, Covariate-adjusted log_10_(p-tau181) levels in the Pavia (**e**), Heidelberg (**f**), Groningen (**g**) and Tübingen (**h**) cohorts. In each cohort, an analysis of covariance (ANCOVA) was performed to examine the effect of disease grouping on log_10_(p-tau181) levels, after adjusting for visit age and sex. There was a significant difference in log_10_(p-tau181) levels by disease group in the Pavia (*F*(1,70) = 30.05, *P* = 3.781 × 10^−10^), Heidelberg (*F*(3,107) = 7.20, *P* = 1.908 × 10^−4^) and Groningen cohorts (*F*(2,61) = 9.03, *P* = 3.68 × 10^−4^). Tukey post-hoc tests (two-sided, multiple comparison corrected) in the Pavia and Groningen cohorts revealed significant differences between the CTRL group and the AL and ATTR groups. In the Heidelberg cohort, only patients with AL reached significance. No difference between the PNP-other group and CTRL group was found in the Heidelberg and Tübingen cohorts. **i**, Combining data across cohorts, covariates-adjusted *z*-scored log_10_(p-tau181) levels across the Pavia (squares), Heidelberg (triangles), Groningen (circles) and Tübingen (diamonds) cohorts. Serum log_10_(p-tau181) levels were standardized (*z*-scored) based on the within-cohort CTRL group to better harmonize levels across cohorts. An ANCOVA was performed to examine the main effect of disease grouping on *z*-scored log_10_(p-tau181) levels, adjusting for visit age and sex. A significant difference in log_10_(p-tau181) levels by disease group (*F*(3,271) = 28.70, *P* = 3.808 × 10^−16^) was observed. Tukey post-hoc tests (two-sided, multiple comparison corrected) revealed significant differences between both the AL and ATTR groups and the CTRL group. Patients in the PNP-other group did not differ on log_10_(p-tau181) levels compared with the CTRL group. **j**, ROC analyses in a multicohort sample comparing the ATTR and AL groups (black), AL group only (cyan) and ATTR group only (magenta) with the CTRL group. Reference is displayed as a gray dashed line. Models include terms for age and sex, and log_10_(p-tau181) levels. All *t* values and *P* values of the post-hoc tests for this figure are given in Extended Data Table 2. AUC, sensitivity and specificity for each comparison are given in Extended Data Table 3. The boxplots depict the median, 25th and 75th quartile, and the whiskers extend to the minimum and maximum. Significant pairwise comparisons are depicted as *P < 0.05, **P < 0.01 and ***P < 0.001.

A total of 280 serum samples from cases of AL and wild-type or variant ATTR amyloidosis, from PNP not due to amyloidosis (‘PNP-other’) and from controls (CTRL) were analyzed. The samples came from four different centers: Pavia (Italy), Heidelberg (Germany), Groningen (the Netherlands) and Tübingen (Germany). Study participants’ demographic and clinical characteristics for each of the four cohorts are summarized in Extended Data Table 1. The age range between groups and cohorts was similar, except for patients with wild-type ATTR (ATTRwt), who were on average older than those with genetic (variant) ATTR (ATTRv). The heart was the most affected organ in both patients with ATTR and those with AL, with a high percentage of patients with AL also exhibiting kidney involvement. Patients with ATTRwt lacked obvious PNP involvement but had more extensive cardiac damage than those with ATTRv, as at least partly reflected by higher serum NT-proBNP and cardiac troponin levels (Extended Data Table 1). Serum p-tau181 levels in the CTRL group were around 0.5–1.0 pg ml^−1^ (for Pavia, Heidelberg and Tübingen), consistent with the concentration in previous reports and the observation that p-tau levels are lower in serum than in plasma^11^. The Groningen cohort revealed lower p-tau181 levels (Fig. 1a–d and Extended Data Table 1) for unknown reasons but perhaps related to differences in handling and long-term storage^12^.

In a first analysis, each cohort was examined separately. For the Pavia, Heidelberg and Groningen cohorts, serum p-tau181 levels differed by disease grouping, after accounting for age and sex (Fig. 1e–h). After accounting for disease grouping and sex, greater age was associated with higher serum p-tau181 levels in the Pavia (*F*(1,70) = 21.94, *P* < 0.001), Heidelberg (*F*(1,107) = 4.32, *P* = 0.040), Groningen (*F*(1,61) = 9.29, *P* = 0.003) and Tübingen (*F*(1,21) = 17.89, *P* < 0.001) cohorts. After accounting for disease grouping and age, being male was associated with higher serum p-tau181 levels in the Pavia cohort (*F*(1,70) = 9.37, *P* = 0.003). All subsequent analyses were adjusted for age and sex. Pairwise comparisons revealed increased p-tau181 levels in AL and ATTR compared to CTRL in the Pavia and Groningen cohorts (Fig. 1e–h and Extended Data Table 2). In the Heidelberg cohort, increased levels of p-tau181 were observed in the AL group compared to CTRL. In addition, in the Pavia cohort, patients with AL had higher p-tau181 levels than those with ATTR (Fig. 1e–h and Extended Data Table 2). When examining the relevance of PNP in the absence of amyloid (that is, ‘PNP-other’) on serum p-tau181 levels, no difference was found in either the Heidelberg or Tübingen cohorts for which these data were available (Fig. 1f,h and Extended Data Table 2).

Next cohorts were combined (*z*-scored) into a larger multicohort sample (Fig. 1i). Again, p-tau181 levels differed across disease groups. Post-hoc analyses revealed between-group differences similar to those observed for the individual sites; that is, the AL and ATTR groups showed increased p-tau181 levels compared to CTRL, while ‘PNP-other’ was not different from CTRL (Fig. 1i and Extended Data Table 2).

Individuals with AL or ATTR can have impaired kidney function, and renal dysfunction has been associated with blood p-tau181 levels^13^. Sensitivity analyses, additionally adjusting for creatinine levels, were performed in a large subset of individuals with available creatinine data (*n* = 266) in the combined multicohort sample. Serum p-tau181 levels differed by disease grouping, after accounting for age, sex and creatinine (*F*(3,257) = 21.502, *P* < 0.001), and between-group post-hoc analyses were not influenced by the inclusion of creatinine in the model; that is, as in the main analyses, AL and ATTR groups showed increased p-tau181 levels compared to CTRL, while PNP-other was not different from CTRL.

In the multicohort sample, p-tau181 discriminated between the CTRL and amyloid (ATTR and AL combined) groups, with an area under the curve (AUC) of 0.82 (Fig. 1j). Youden’s index provided a threshold of 0.68, which yielded a sensitivity of 0.75 and a specificity of 0.80. Subsequent analyses compared CTRL versus AL alone and CTRL versus ATTR alone and found similarly good discrimination (AUC of 0.85 and 0.83, respectively) (Fig. 1j and Extended Data Table 3). For individual sites, p-tau181 discriminated between CTRL and amyloid (ATTR and AL combined) with an AUC of 0.94 (Pavia), 0.74 (Heidelberg) and 0.81 (Groningen).

**Extended Data Fig. 1:**
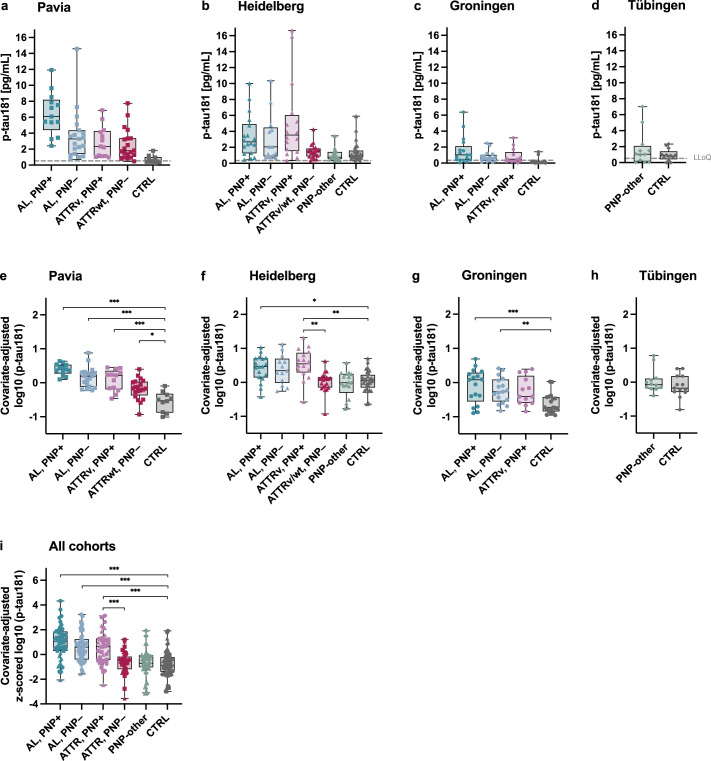
Influence of PNP status on serum p-tau181 levels in AL and ATTR patients from four different cohorts. Absolute (non-transformed) serum p-tau181 levels for AL and ATTR patients (subgrouped into AL,PNP + , AL,PNP- and ATTR,PLP+ and ATTR,PLP-), PNP-other and CTRL for the (**a**) Pavia cohort, (**b**) Heidelberg cohort, (**c**) Groningen cohort, and (**d**) Tübingen cohort. For each cohort the lower functional Limit of Quantification (LLoQ) is indicated by a gray dashed line. Covariate-adjusted log_10_(p-tau181) levels within (**e**) Pavia, (**f**) Heidelberg, (**g**) Groningen, and (**h**) Tübingen cohorts. Within each cohort, an ANCOVA was performed to examine the effect of disease grouping on log_10_(p-tau181) levels, after adjusting for visit age and sex. There was a significant difference in log_10_(p-tau181) levels by disease group within Pavia (F(4,68) = 20.93, p = 2.8 x 10^−11^), Heidelberg (F(5,105) = 7.76, p = 3.1 x 10^−6^), and Groningen cohorts (F(3,60) = 6.24, p = 9.23 x10^−4^). Tukey post-hoc tests (two-sided, multiple comparison corrected) within the Pavia cohorts revealed significant elevated p-tau181 levels between CTRL and all AL and ATTR groups. Within the Heidelberg cohort, AL,PNP+ and AL,PNP+ revealed significant differences to CTRL. Additionally, ATTR,PNP+ patients had higher levels than ATTR,PNP-. The Groningen cohort, revealed significant differences between both AL,PNP+ and AL,PNP- and CTRL patients. The t-value and p-values of the post-hoc tests are given in Extended Data Table 2. (**i**) Combing data across cohorts, covariates-adjusted z-scored log_10_(p-tau181) levels across the Pavia (squares), Heidelberg (triangles), Groningen (circles), and Tübingen (diamond) cohorts are presented. Serum log_10_(p-tau181) levels were standardized (z-scored) based on the within-cohort CTRL group to harmonize levels across cohorts. An ANCOVA was performed to examine the main effect of disease grouping on z-scored log_10_(p-tau181) levels, adjusting for visit age and sex. A significant difference in log_10_(p-tau181) levels by disease group (F(5,269) = 23.202, p = 2.29 x 10^−19^) was observed. Tukey post-hoc tests (two-sided, multiple comparison corrected) revealed significant differences between all amyloid groups and CTRL, with the exception of the ATTR,PNP- group. Moreover, ATTR,PNP+ patients had significantly higher p-tau levels compared to ATTR,PNP- patients. No difference between patients with PNP but without concomitant amyloid and CTRL was found. T-value and p-values of the post-hoc tests are given in Extended Data Table 2. The boxplots depict the median, 25^th^ and 75^th^ quartile and the whiskers extend to the minimum and maximum. Significant pairwise comparisons are depicted as * for p < 0.05, ** for p < 0.01, and *** for p < 0.001.

Because PNP is also a common clinical finding in patients with AL or ATTR (Extended Data Table 1), we further explored whether the presence of PNP is contributing to the elevated p-tau181 levels in patients with AL or ATTR. We subset our patients with AL or ATTR into those with PNP (PNP+) and those without PNP (PNP−) (Extended Data Fig. 1a–d). For the Pavia cohort, pairwise comparisons with CTRL revealed increased levels of p-tau181 in the AL,PNP+ and AL,PNP− as well as ATTR,PNP+ and ATTR,PNP− subgroups. In the Heidelberg cohort, only the AL,PNP+ and ATTR,PNP+ groups reached significance, while in the Groningen cohort, AL,PNP+ and AL,PNP− reached significance (Extended Data Fig. 1e–h and Extended Data Table 2). When cohorts were combined (*z*-scored) post-hoc analyses revealed between-subgroup differences similar to those observed for the individual cohorts; that is, patients with AL showed increased p-tau181 levels independent of PNP, whereas p-tau levels in patients with ATTR were increased only in the presence of PNP (Extended Data Fig. 1i and Extended Data Table 2).

**Extended Data Fig. 2:**
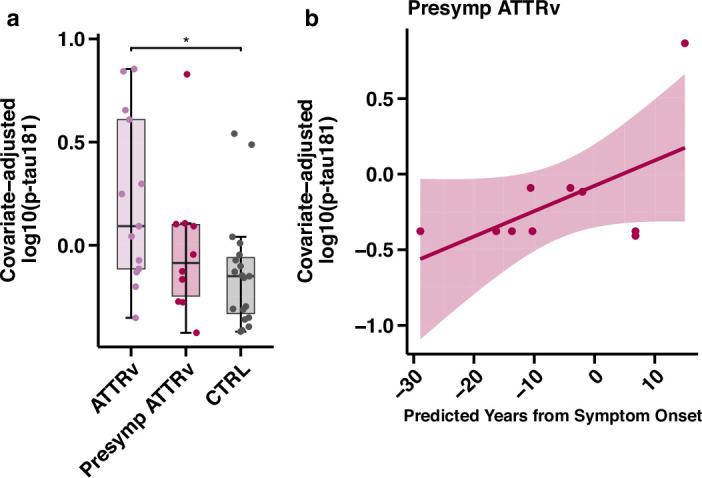
Serum p-tau181 levels for ATTRv, presymptomatic ATTRv, and CTRL in the Groningen cohort. (**a**) Covariate (age, sex)-adjusted serum log_10_(p-tau181) levels within the Groningen cohort. Patients were grouped based on disease status: ATTRv = patients with genetic (variant) transthyretin amyloidosis; PreSymp ATTRv = ATTRv patients in the presymptomatic stages of disease; CTRL = Controls. An ANCOVA was performed to examine the effect of disease grouping on log_10_(p-tau181) levels, after adjusting for visit age and sex. There was a significant difference in log_10_(p-tau181) levels by disease group (F(2, 37) = 3.99, p = 0.027). Tukey post-hoc tests (two-sided, multiple comparison corrected) revealed significant differences between ATTRv and CTRL (as reported in Fig. 1c). The Presymp ATTRv did not significantly differ from the ATTRv (p = 0.325) or the CTRL (p = 0.692) groups. Significant pairwise comparisons are depicted as * for p < 0.05. The boxplots depict the median, 25^th^ and 75^th^ quartile and the whiskers extend to 1.5 x interquartile range (IQR). (**b**) Linear regression revealed that covariate (sex)-adjusted serum log_10_(p-tau181) levels increased as a function of years from predicted symptom onset in presymptomatic ATTRv carriers (B [SE] =0.02 [0.01], p = 0.040). For the calculation of predicted years from symptom onset, see Methods. The shaded area around the linear fit line represents the 95% confidence interval.

To examine whether p-tau181 levels had already increased at asymptomatic stages, a further small cohort of presymptomatic ATTR mutation carriers (*n* = 10 from the Groningen site; Methods) was studied. Increased p-tau181 levels were already found at this presymptomatic stage and were associated with the predicted years from symptom onset (Extended Data Fig. 2).

**Extended Data Fig. 3:**
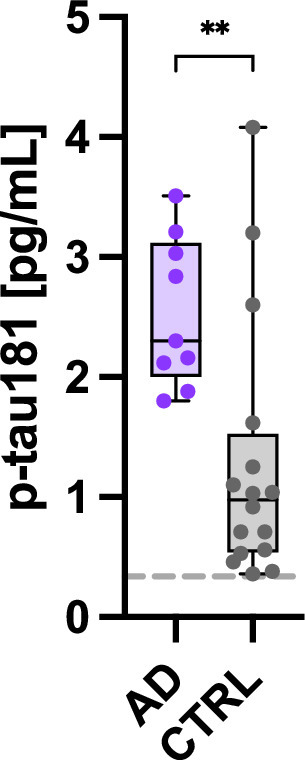
Serum p-tau181 levels in AD patients. Serum p-tau181 levels for symptomatic autosomal dominant Alzheimer (AD) patients (n = 9) and age-matched non-mutation controls (CTRL) (n = 16). Box plots with median and IQR (whiskers extend to the minimum and maximum) with the lower limit of quantification (LLoQ=0.338 pg/ml; gray dashed line) are shown. Two-tailed t-test of log_10_(p-tau181): t(23 = 3.647; p = 0.0013. Note that the fold increase between AD and CTRL is 2.4, similar to or less than that found in ATTR and AL patients compared to CTRL (Fig. 1; Extended Data Table 1).

For a direct comparison of serum p-tau181 elevations in the ATTR and AL groups with serum p-tau181 levels in AD, yet another small cohort (from the Tübingen site) of patients with symptomatic AD (*n* = 9) versus CTRL (n = 16) was studied (Methods). Results revealed a median (interquartile range (IQR)) of 2.30 (2.00–3.12) pg ml^−1^ for the AD group compared with a median (IQR) of 0.98 (0.54–1.53) pg ml^−1^ for the CTRL group; a 2.4-fold increase (Extended Data Fig. 3). This increase is similar to the absolute levels and fold increases of serum p-tau181 observed in patients with AL or ATTR (Fig. 1a–d and Extended Data Table 1) and less compared with the patients with amyloid neuropathy (AL,PNP+; ATTR,PNP+) (Extended Data Fig. 1a–d).

**Fig. 2:**
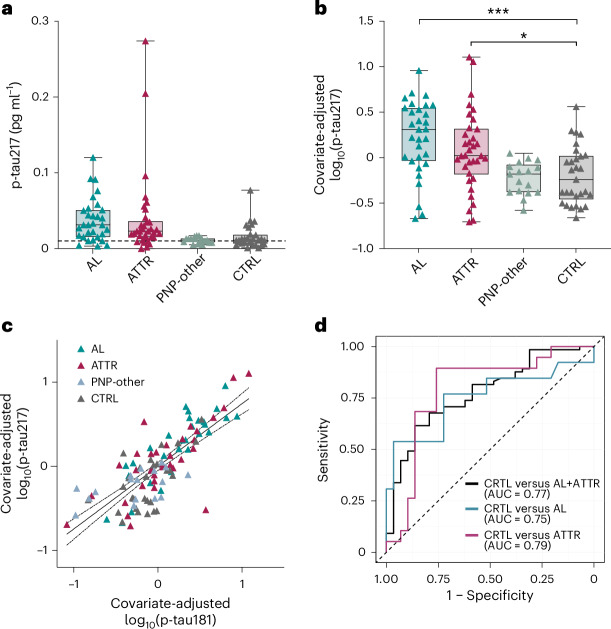
Serum p-tau217 levels for AL and ATTR groups from the Heidelberg cohort. **a**, Absolute (nontransformed) serum p-tau217 levels in AL and ATTR groups from the Heidelberg cohort. Patient characteristics and numbers of patients are given in Extended Data Table 1. Patients were grouped based on disease status. Lower functional LLoQ is indicated by a gray dashed line. **b**, Covariate-adjusted log_10_(p-tau217) levels. An ANCOVA was performed and after adjusting for visit age and sex there was a significant difference in log_10_(p-tau217) levels by disease group (*F*(3,105) = 9.17, *P* = 1.931 × 10^−5^). Tukey post-hoc tests (two-sided, multiple comparison corrected) revealed significant differences between CTRL and both patients with AL (*t* = −4.339; P = 1.61 × 10^−4^) and those with ATTR (*t* = −2.855; *P* = 0.026). **c**, Spearman correlation (two-sided) of visit age- and sex-adjusted serum log_10_(p-tau217) and log_10_(p-tau181) levels revealed a significant association (Spearman’s ϱ = 0.761; *P* = 3.32 × 10^−22^). **d**, ROC analyses comparing the ATTR and AL groups (black), AL group only (cyan) and ATTR group only (magenta) with the CTRL group. Reference is displayed as a gray dashed line. Models include terms for age and sex, and log_10_(p-tau217) levels. Detailed numbers for AUC, sensitivity and specificity for each comparison are presented in Extended Data Table 4. The boxplots depict the median, 25th and 75th quartile and the whiskers extend to the minimum and maximum. Significant pairwise comparisons are depicted as *P < 0.05, **P < 0.01 and ***P < 0.001.

Finally, remaining serum samples of the Heidelberg cohort were used for p-tau217 measurements to determine whether the increase in p-tau seen for patients with AL and ATTR was specific to the p-tau181 phosphorylation site or also occurred at other tau sites such as p-tau217. Despite much lower absolute levels of serum p-tau217 than p-tau181, the relative increase in p-tau217 was comparable with that of p-tau181 (Fig. 2a and Extended Data Table 1). In contrast to p-tau181, increased levels for covariate-adjusted p-tau217 also reached significance for patients with ATTR in the Heidelberg cohort (Fig. 2b). There was also a significant association between p-tau181 and p-tau217 levels (Fig. 2c). Similar to our analyses with p-tau181 for all sites combined and p-tau181 for the Heidelberg cohort, p-tau217 showed good discrimination between the CTRL and amyloid (ATTR and AL combined) groups, with an AUC of 0.77 (Fig. 2d). Subsequent analyses that compared CTRL versus AL alone and CTRL versus ATTR alone revealed AUCs of 0.75 and 0.79, respectively (Fig. 2d and Extended Data Table 4).

## Discussion

Our results show robust increases in p-tau181 and p-tau217 levels in the blood of people with AL and ATTR amyloidosis. The increase was more pronounced in the presence of PNP. However, and importantly, p-tau181 and p-tau217 were not elevated in PNP unrelated to amyloidosis (PNP-other). This suggests that the cause of elevated p-tau levels in people with ATTR and AL was the amyloid, and that people with ATTR and AL with PNP are likely to be at a more advanced stage of the disease.

Phosphorylated tau181 in blood is a well-established fluid biomarker for Aβ deposition in AD and is now being considered for routine assessment to identify preclinical AD stages and to stratify patients for early Aβ immunotherapy treatment^14–16^. Both p-tau181 and p-tau217 increase in blood before neuronal damage (indicated by an increase in neurofilament light chain) or tau pathology (positron emission tomography imaging in humans, histopathology in mouse models) can be detected^6,7,10^. Consistently, it has been suggested that phosphorylation of tau in early stages of AD is the result of an amyloid-induced stress response of neurons in the vicinity of Aβ plaques^17–19^, whereas in later stages of AD, elevated p-tau drives tauopathy^20^.

Tau is expressed not only in the brain, but also in peripheral organs and tissues affected by AL and ATTR, such as peripheral nerves^21^, cardiomyocytes and skeletal muscle^22,23^, or renal podocytes^24^. It is conceivable that amyloid deposition in these tissues also promotes an amyloid-induced cellular stress response with release of hyperphosphorylated tau into the blood.

The AL and ATTR blood samples used here were taken from untreated people with no clinical signs of dementia. Early asymptomatic AD changes in people with AL or ATTR cannot be excluded, but the same is true for the controls, who were of a very similar age. Moreover, there is no evidence in the literature suggesting that people with ATTR or AL are more prone to developing AD. A positive correlation between cardiac ATTR deposition and AD brain pathology has been reported at autopsy, but this was attributed to shared lifestyle-related risk factors for both conditions^25^. Some people with ATTRv also exhibit ATTR deposition in leptomeningeal vessels^26^. For these individuals, some of the blood p-tau may originate from ATTR deposition in the central nervous system. For people with AL, renal dysfunction (renal amyloid involvement was found in about half of the individuals) may have contributed to the elevated p-tau levels in blood^27,28^. However, sensitivity analysis adjusting for creatinine levels did not influence the current results.

The ability of p-tau181 and p-tau217 to distinguish patients with ATTR and those with AL from controls, as demonstrated by their robust AUC values in receiver operating characteristic (ROC) analyses, supports their potential as biomarkers reflecting systemic amyloid pathology. These findings may complicate blood-based presymptomatic AD diagnosis because p-tau181 and p-tau217 elevations in AL and ATTR are in the same range (two- to threefold) as for AD^15,29^. However, tau is expressed in the periphery as a high molecular weight 110-kDa protein isoform (‘big tau’^21,22^) and it is conceivable that developing p-tau assays specific for the high molecular weight tau isoforms^28,30^ may play an important role in validating tau immunoassays for AD, and have the potential to increase their specificity for AL and ATTR amyloid. Such assays will further facilitate p-tau in blood as a diagnostic tool for ATTR and AL amyloidosis, as well as for the clinical assessment and differential diagnosis of PNP etiologies.

## Methods

### Clinical cohorts

We analyzed serum samples from cases of AL and ATTR amyloidoses with or without PNP. In addition, serum samples from patients with PNP not due to amyloidosis (termed ‘PNP-other’) and controls (CTRL) were analyzed. The samples came from four different centers: Pavia (Italy), Heidelberg (Germany), Groningen (the Netherlands) and Tübingen (Germany) (Extended Data Table 1). ATTR was either sporadic (ATTRwt) or genetic (ATTRv). Clinical diagnosis was done using standard guidelines^31,32^. In patients with AL, PNP was defined as symmetric distal neuropathic symptoms or signs of sensory loss. For PNP assessment in patients with ATTR, disease severity was scored according to the Coutinho staging system^33^. The CTRL group had no signs of ATTR or AL amyloidoses and no signs of PNP. Detailed study participant characteristics are given in Extended Data Table 1.

In addition, and not included in Extended Data Table 1, there were serum samples (*n* = 10) from presymptomatic ATTRv (from the Groningen site) with ‘predicted years from symptom onset’. Predicted years from symptom onset were calculated based on family history, a combination of ‘predicted years from symptom onset’ reported in the literature (based on the genetic variant) and family history, or if no first-degree family member was known, only from the literature. In addition, serum samples from patients with symptomatic AD with an autosomal-dominant mutation (*n* = 9; 53.8 ± 7.8 years of age, mean ± s.d.) and from control nonmutation family members (*n* = 16; 41.2 ± 9.9 years of age, mean ± s.d.) were used (from the Tübingen site).

Informed consent was obtained from all research participants. Ethics approval for the study was obtained from the ethics committee at the medical faculty of the University of Tübingen (442/2024BO2 and 1017/2020BO2), from Heidelberg (123/2006), from Groningen (UMCG registration number 17395) and from Pavia (local institutional review board approval: N.20190103452 and N.20200045840).

### Serum sampling and p-tau181 measurement

For the Pavia cohort, venipuncture blood samples were centrifuged at 2,200 *g* for 10 min at room temperature, and serum stored at −80 °C within about 3 h after collection. For the Heidelberg cohort, venipuncture blood samples were centrifuged at 2,500 *g* for 15 min at room temperature and serum stored at −80 °C within about 3 h after collection. For the Groningen cohort, venipuncture blood samples were centrifuged at 1,255 *g* for 10 min at room temperature and serum stored at −20 °C within 1 h until further storage at −80 °C within several months of collection. For the Tübingen cohort, after venipuncture blood samples were centrifuged at 2,000 *g* for 10 min at 4 °C and serum was stored at −80 °C within 90 min after collection.

Samples from Pavia, Heidelberg and Groningen were shipped to Tübingen on dry ice and stored at −80 °C until analysis. On the day of analysis, serum samples were thawed on wet ice for 1 h. Afterwards they were vortexed for 30 s (VortexGenie2 at level 5 ~1,800 rpm) and centrifuged for 5 min at 10,000 *g* at 4 °C. For p-tau181 measurements, two commercially available assay kits were used (p-tau181 V2 Advantage Kit (cat. no. 103714) and the follow-up version p-tau181 V2.1 Advantage Kit (cat. no. 104111)). All samples were measured on the Simoa HD-X platform (Quanterix). Serum samples were 1:4 auto-diluted with p-tau181 sample diluent. Interassay variability was evaluated with three native human CSF samples.

To match the p-tau181 V2 Advantage Kit measurements (including the corresponding coefficient of variation (CV)) to the most recent assay version (p-tau181 V2.1) the measurements were adjusted according to the manufacturer’s recommendation: $y={\left(\frac{-783.1x}{(x-8,993)}+\frac{1,293}{(x-8,993)}\right)}^{1.093}$. If the concentrations were below the functional LLoQ, the values were imputed $\left(\frac{\text{functional LLoQ}}{2}\right)$. The functional LLoQ for the V2 version was 0.338 pg ml^−1^ and for the V2.1 version was 0.524 pg ml^−1^ (that is, 8 pg ml^−1^ according to the manufacturer and 0.524 pg ml^−1^ after conversion with the formula). Although for Pavia, Heidelberg and Tübingen values below the LLoQ were restricted to CTRL and ‘PNP-other’ (with one and two exceptions for Pavia and Heidelberg, respectively), mean p-tau181 values for the Groningen samples were systematically lower than the concentrations measured in the other cohorts with almost 50% of samples below LLoQ, again the majority being CTRL samples. Some Groningen samples did not reveal any p-tau181 values (largely CTRL individuals) and were not included in the analysis (and are not included in Extended Data Table 1).

All samples were measured in duplicate in a blinded manner. From three samples only one technical replicate was obtained, and the single measurement was taken for further analysis. From all other samples the mean of the duplicates was taken. From ten samples with values above LLoQ the CV of the duplicate measurement was >25% (that is, <5% of all samples). One sample was excluded from the Heidelberg cohort because it was a significant outlier for p-tau181, p-tau217 and also for the creatinine measurements (Grubb’s test).

### p-tau217 measurement

In the Heidelberg cohort, except for one sample, there was enough serum for additional p-tau217 measurements (for some of these samples an additional freeze–thaw cycle was necessary). One other sample was excluded because it was a significant outlier for p-tau181, for p-tau217 and also for creatinine measurements (Grubb’s test). The commercially available Advantage PLUS Kit (cat. no. 104588) on the Simoa HD-X platform (Quanterix) was used. Serum samples were auto-diluted 1:2 with p-tau217 sample diluent and measured in duplicates, blinded. The mean of the duplicates was taken. For four samples only one technical replicate was obtained, and the single measurement was taken for further analysis. Seven samples above the LLoQ had a CV of the duplicate >25% (that is 6.1% of all samples). One sample was excluded because it was an outlier for p-tau217, p-tau181 and also for the creatinine measurements. One sample did not reveal any values and was not included in the analysis. If concentrations were below the functional LLoQ (0.01 pg ml^−1^), the values were imputed $\left(\frac{\text{funtional LLoQ}}{2}=0.005\,{\rm{pg}}\,{\rm{ml}}^{-1}\right)$.

### Statistical analysis

Absolute (nontransformed) serum p-tau181 levels for all individuals, across all cohorts and disease groups (AL, ATTR, PNP-others, CTRL) and (AL,PNP+, AL,PNP−, ATTR,PNP+, ATTR,PNP−, PNP-others, CTRL) are shown in Fig. 1a–d and Extended Data Fig. 1a–d, respectively. For statistical analysis all values below LLoQ were imputed. Across cohorts, a Shapiro–Wilk test (stats package in R) indicated that p-tau181 levels deviated significantly from normality, *W*(290) = 0.66, *P* < 2.2 × 10^−16^ and a log_10_ transformation was applied before analyses.

In each cohort separately, an ANCOVA (stats package in R), adjusting for visit age and sex, was performed to examine whether disease grouping was associated with serum log_10_(p-tau181) levels. Tukey post-hoc analyses (multcomp package in R) were performed to further examine pairwise comparisons between: (1) the CTRL group and all available disease groups in each cohort, and (2) the AL group and the ATTR group.

To examine whether disease grouping was associated with serum log_10_(p-tau181) levels in a larger multicohort sample, harmonized *z*-scored serum log_10_(p-tau181) data (based on the mean and s.d. of the CTRL group in each cohort) were combined across all cohorts. ANCOVA analysis, adjusting for visit age and sex, was performed. Subsequent Tukey post-hoc analyses were performed to further examine pairwise comparisons between: (1) the CTRL group and all available disease groups in each cohort, and (2) the AL group and the ATTR group. In addition, we performed ROC analyses (pROC package in R) in multicohort sample comparing: (1) the ATTR and AL groups, (2) the AL group only and (3) the ATTR group only with the CTRL group. Models include terms for age, sex and log_10_(p-tau181) levels.

Next, as a sensitivity analysis, we additionally adjusted for creatinine levels in a subset of individuals with available data (*n* = 266) in the combined multicohort sample. In addition, to explore the effect of PNP on the observed associations, we further subset our AL and ATTR groups into those with and without PNP and repeated our main analyses.

For the Heidelberg cohort, a separate ANCOVA was performed, adjusting for visit age and sex, to examine whether disease grouping was associated with serum log_10_(p-tau217) levels. Again, a Shapiro–Wilk test indicated that the p-tau217 levels deviated significantly from normality, *W*(111) = 0.59, *P* = 3.43 × 10^−16^ and a log_10_ transformation was applied before analyses. Tukey post-hoc analyses were performed to further examine pairwise comparisons between the CTRL group and all available disease groups in this cohort. Subsequently, we examined the association between log_10_(p-tau217) and log_10_(p-tau181) measurements in this cohort using a linear regression analysis, after adjusting for age and sex. Similar to our analyses with p-tau181, we performed ROC analyses sample comparing: (1) the ATTR and AL groups, (2) the AL group only and (3) the ATTR group only with the CTRL group. Models include terms for age, sex and log_10_(p-tau217) levels.

For the Groningen cohort, a separate ANCOVA was performed, adjusting for age and sex, to examine whether patients with ATTRv (in both symptomatic and presymptomatic stages) had higher levels of log_10_(p-tau181) compared with the CTRL group, after adjusting for age and sex. Tukey post-hoc analyses were performed to further examine pairwise comparisons between the CTRL group and (1) presymptomatic ATTRv carriers and (2) symptomatic ATTRv carriers. We additionally examined the association between predicted years from symptom onset and log_10_(p-tau181) using a linear regression analysis, adjusting for sex. Each patient’s predicted year from symptom onset was calculated by taking their visit age minus their estimated year of symptom onset.

Finally, levels of serum p-tau181 for AD versus CTRL (Extended Data Fig. 3) were compared using the *t*-test of log_10_-transformed values. Analyses were performed in R (v.4.4.2) and GraphPad Prism (v.10.6.1).

### Reporting summary

Further information on research design is available in the Nature Portfolio Reporting Summary linked to this article.

## Online content

Any methods, additional references, Nature Portfolio reporting summaries, source data, extended data, supplementary information, acknowledgements, peer review information; details of author contributions and competing interests; and statements of data and code availability are available at 10.1038/s41591-026-04272-2.

## Supplementary information

Reporting Summary

## Figures and Tables

**Extended Data Table 1:**
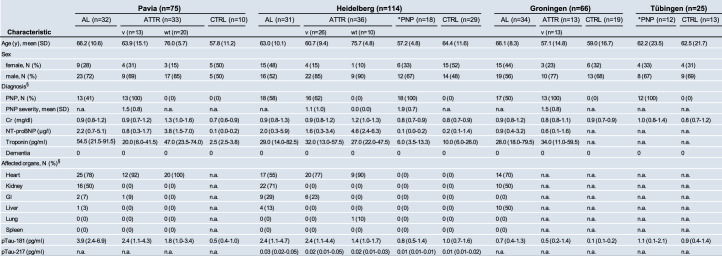
Characteristics of cohort samples

*PNP due to: vitamin B12 deficiency (*n* = 2), monoclonal gammopathy (*n* = 3); diabetes (*n* = 1); auto-inflammation (*n* = 3); Immunoglobulin M (IgM) anti-myelin-associated glycoprotein (*n* = 1); endocrinological disorder (*n* = 1); hereditary (*n* = 1); unknown causes (*n* = 18).

§Based on the total number of patients/samples for which this information was available.

CR, creatinine; GI, gastrointestinal tract; Troponin (high-sensitive troponin I for Pavia; high-sensitive troponin T for Heidelberg and Groningen).

If not otherwise indicated, median values and IQR range are presented.

**Extended Data Table 2:**
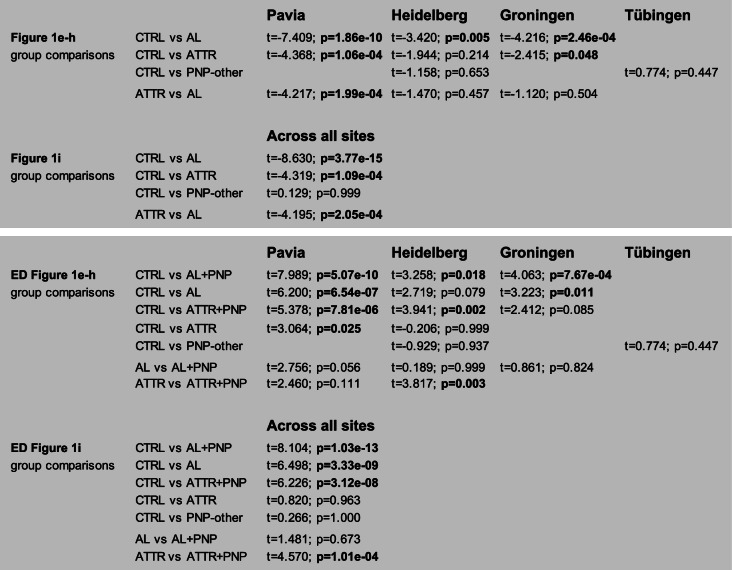
Pairwise comparisons of p-tau181 levels across patient groups from four different cohorts

**Extended Data Table 3:**
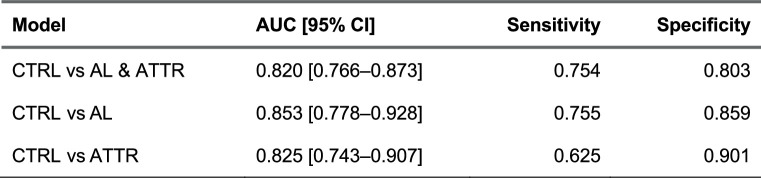
ROC analysis of p-tau181 in multi-cohort sample

ROC analysis in multi-cohort sample was used to assess group-level discriminatory capability of p-tau181. The area under the ROC curve (AUC) from each comparison was used to assess the performance of log10(p-tau181) levels in combination with age and sex in discriminating between amyloid groups (AL + ATTR, AL alone, and ATTR alone) and control group (CTRL). AUC with 95% confidence intervals (calculated with DeLong method), specificity and sensitivity are reported.

**Extended Data Table 4:**
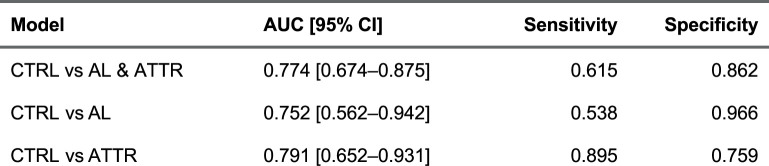
ROC analysis of p-tau217 in the Heidelberg cohort

ROC analysis for the Heidelberg samples was used to assess group-level discriminatory capability of p-tau217. The area under the ROC curve (AUC) from each comparison was used to assess the performance of log_10_(p-tau217) levels in combination with age and sex in discriminating between amyloid groups (AL + ATTR, AL alone, and ATTR alone) and control group (CTRL). AUC with 95% confidence intervals (calculated with DeLong method), specificity and sensitivity are reported.

## Data Availability

The data used for this study will be shared with qualified investigators for the purpose of replicating the results of this study. Requests should be made to the corresponding authors. These requests will be reviewed to ensure confidentiality and compliance with EU legislation on general data protection. These procedures are designed to safeguard participant anonymity and ensure that data is only used in accordance with the terms set out in the IRB approvals.

## Code availability

The R source code used in this study is available via GitHub at https://github.com/stephaschultz/pTau_amyloid_neuropathies.
