# Supplementary material for: Blood phosphorylated tau elevation as a biomarker in immunoglobulin light chain and transthyretin amyloidosis
Source: Nat Med. 2026 Mar 11;32(5):1676–81. doi: 10.1038/s41591-026-04272-2 (PMC13190286; doi:10.1038/s41591-026-04272-2)
Supplement: Supplementary file 1 — Reporting Summary [file 41591_2026_4272_MOESM1_ESM.pdf]

nature portfolio

Corresponding author(s): Mathias Jucker, Stefan Schönland, Hans Nienhuis, Giovanni Palladini

Last updated by author(s): Nov 7, 2025

Reporting Summary

Nature Portfolio wishes to improve the reproducibility of the work that we publish. This form provides structure for consistency and transparency in reporting. For further information on Nature Portfolio policies, see our Editorial Policies and the Editorial Policy Checklist.

Statistics

For all statistical analyses, confirm that the following items are present in the figure legend, table legend, main text, or Methods section.

n/a	Confirmed	
☐	☒	The exact sample size (*n*) for each experimental group/condition, given as a discrete number and unit of measurement
☐	☒	A statement on whether measurements were taken from distinct samples or whether the same sample was measured repeatedly
☐	☒	The statistical test(s) used AND whether they are one- or two-sided<br>*Only common tests should be described solely by name; describe more complex techniques in the Methods section.*
☐	☒	A description of all covariates tested
☐	☒	A description of any assumptions or corrections, such as tests of normality and adjustment for multiple comparisons
☐	☒	A full description of the statistical parameters including central tendency (e.g. means) or other basic estimates (e.g. regression coefficient) AND variation (e.g. standard deviation) or associated estimates of uncertainty (e.g. confidence intervals)
☐	☒	For null hypothesis testing, the test statistic (e.g. *F*, *t*, *r*) with confidence intervals, effect sizes, degrees of freedom and *P* value noted<br>*Give P values as exact values whenever suitable.*
☒	☐	For Bayesian analysis, information on the choice of priors and Markov chain Monte Carlo settings
☒	☐	For hierarchical and complex designs, identification of the appropriate level for tests and full reporting of outcomes
☐	☒	Estimates of effect sizes (e.g. Cohen's *d*, Pearson's *r*), indicating how they were calculated

Our web collection on statistics for biologists contains articles on many of the points above.

Software and code

Policy information about availability of computer code

Data collection	No software was used.
Data analysis	Analyses of Figs. 1 and 2 and Extened Data Fig 1and 2 were performed in R. source code available, with publication, at https://github.com/stephaschultz/pTau_amyloid_neuropathies. Extended Data Fig. 3 was done using Graph Pad Prism Version 10.5.0.

For manuscripts utilizing custom algorithms or software that are central to the research but not yet described in published literature, software must be made available to editors and reviewers. We strongly encourage code deposition in a community repository (e.g. GitHub). See the Nature Portfolio guidelines for submitting code & software for further information.

Data

Policy information about availability of data

All manuscripts must include a data availability statement. This statement should provide the following information, where applicable:

- Accession codes, unique identifiers, or web links for publicly available datasets
- A description of any restrictions on data availability
- For clinical datasets or third party data, please ensure that the statement adheres to our policy

The data used for this study will be shared with qualified investigators for the purpose of replicating the results of this study. Requests should be made to the corresponding authors. These requests will be reviewed to ensure confidentiality and compliance with EU legislation on general data protection. These procedures are designed to safeguard participant anonymity and ensure that data is only used in accordance with the terms set out in the IRB approvals.

Research involving human participants, their data, or biological material

Policy information about studies with human participants or human data. See also policy information about sex, gender (identity/presentation), and sexual orientation and race, ethnicity and racism.

Reporting on sex and gender	In our demographic breakdown of the study data, we have included percentage of sample reported as female and males to describe the distributions of biological sex within our cohorts. Sex was considered and included as a covariate in models.
Reporting on race, ethnicity, or other socially relevant groupings	n.a.
Population characteristics	Please see also table 1 of our manuscript. The covariate relevant characteristics of our sample were the following: age, sex, and cohort.
Recruitment	Participants were recruited from regular visits to our hospitals
Ethics oversight	Ethics approval was obtained from the ethics committee at the medical faculty of the University of Tübingen (442/2024BO2 and 1017/2020BO2); from Heidelberg (123/2006); from Groningen (UMCG registration number 17395) and from Pavia (local IRB approval: N.20190103452 and N.20200045840).

Note that full information on the approval of the study protocol must also be provided in the manuscript.

Field-specific reporting

Please select the one below that is the best fit for your research. If you are not sure, read the appropriate sections before making your selection.

☒ Life sciences ☐ Behavioural & social sciences ☐ Ecological, evolutionary & environmental sciences

For a reference copy of the document with all sections, see nature.com/documents/nr-reporting-summary-flat.pdf

Life sciences study design

All studies must disclose on these points even when the disclosure is negative.

Sample size	A total of n=280 serum samples from AL and ATTR (wildtype and mutated/variant) amyloidosis cases with or without polyneuropathy (PNP), from PNP cases not due to amyloidosis, and from controls (CTRL) cases were analyzed. The samples came from four different centers: Pavia (Italy), Heidelberg (Germany), Groningen (Netherlands), and Tübingen (Germany). In addition, serum samples (n=10) from presymptomatic ATTRv cases from the Groningen were used. Moreover, serum samples from AD patients (n=9) and from control patients (n=16; were used from the Tübingen site.
Data exclusions	No date were excluded except one oulier (Fig. 2) defined by the Grubb´s Test (described in Figure legend)
Replication	All samples were measured in duplicate except for a few samples outlined in the method section where only one technical replicate was obtained.
Randomization	There were no experimental groups within our study.
Blinding	All samples were measured in a blinded manner.

Reporting for specific materials, systems and methods

We require information from authors about some types of materials, experimental systems and methods used in many studies. Here, indicate whether each material, system or method listed is relevant to your study. If you are not sure if a list item applies to your research, read the appropriate section before selecting a response.

Materials & experimental systems

n/a	Involved in the study	
☐	☒	Antibodies
☒	☐	Eukaryotic cell lines
☒	☐	Palaeontology and archaeology
☒	☐	Animals and other organisms
☐	☒	Clinical data
☒	☐	Dual use research of concern
☒	☐	Plants

Methods

n/a	Involved in the study	
☒	☐	ChIP-seq
☒	☐	Flow cytometry
☒	☐	MRI-based neuroimaging

Antibodies

Antibodies used	For pTau181 measurements, two commercially available assay kits were used (pTau-181 V2 Advantage Kit Cat 103714 and the follow-up version pTau-181 V2.1 Advantage Kit Cat 104111). For ptau217 the commercially available Advantage PLUS Kit (Cat. 104588) on the Simoa HD-X platform (Quanterix) was used.
Validation	These were ready-to-use and already validated antibodies.

Clinical data

Policy information about clinical studies

All manuscripts should comply with the ICMJE guidelines for publication of clinical research and a completed CONSORT checklist must be included with all submissions.

Clinical trial registration	*Provide the trial registration number from ClinicalTrials.gov or an equivalent agency.*
Study protocol	*Note where the full trial protocol can be accessed OR if not available, explain why.*
Data collection	*Describe the settings and locales of data collection, noting the time periods of recruitment and data collection.*
Outcomes	Serum p-tau 181 and p-tau 217

Plants

Seed stocks	na
Novel plant genotypes	na
Authentication	na
